# Consistency of compact and extended models of glucose-insulin homeostasis: The role of variable pancreatic reserve

**DOI:** 10.1371/journal.pone.0211331

**Published:** 2019-02-15

**Authors:** Andrea De Gaetano, Claudio Gaz, Simona Panunzi

**Affiliations:** 1 CNR-IASI BioMatLab, Consiglio Nazionale delle Ricerche, Istituto di Analisi dei Sistemi ed Informatica, Laboratorio di Biomatematica (Italian National Research Council - Institute for System Analysis and Computer Science - Biomathematics Laboratory), UCSC Largo A. Gemelli 8, Rome, Italy; 2 Sapienza Università di Roma, Dipartimento di Ingegneria Informatica, Automatica e Gestionale (DIAG) (Department of Computer, Control and Management Engineering), Via Ariosto 25, Rome, Italy; Mahidol University, THAILAND

## Abstract

Published compact and extended models of the glucose-insulin physiologic control system are compared, in order to understand why a specific functional form of the compact model proved to be necessary for a satisfactory representation of acute perturbation experiments such as the Intra Venous Glucose Tolerance Test (IVGTT). A spectrum of IVGTT’s of virtual subjects ranging from normal to IFG to IGT to frank T2DM were simulated using an extended model incorporating the population-of-controllers paradigm originally hypothesized by Grodsky, and proven to be able to capture a wide array of experimental results from heterogeneous perturbation procedures. The simulated IVGTT’s were then fitted with the Single-Delay Model (SDM), a compact model with only six free parameters, previously shown to be very effective in delivering precise estimates of insulin sensitivity and secretion during an IVGTT. Comparison of the generating, extended-model parameter values with the obtained compact model estimates shows that the functional form of the nonlinear insulin-secretion term, empirically found to be necessary for the compact model to satisfactorily fit clinical observations, captures the pancreatic reserve level of the simulated virtual patients. This result supports the validity of the compact model as a meaningful analysis tool for the clinical assessment of insulin sensitivity.

## Introduction

Mathematical models for representing and simulating insulin/glucose metabolism, both in normal conditions and under perturbation tests, have been developed and used since the 1960’s [1]. Their importance resides in the possibility to better understand the pathophysiology and development of Type 2 Diabetes Mellitus (T2DM) and of its pre-conditions, such as Impaired Glucose Tolerance (IGT) or Impaired Fasting Glucose (IFG). More extended models are also used to simulate virtual patients in order to test control algorithms, (i.e. [2–6] and references within) in situations where experimental procedures are very expensive or cannot be performed for ethical or practical reasons. Models can be more or less complex according to how many sub-components of the entire metabolic system are simulated (glucagon, adrenalin, Free Fatty Acids, etc.) and according to the level at which the relevant physiology is modelled (e.g. whole-body or cellular). Comprehensive models of the glucose-insulin system [7, 8] allow to simulate healthy subjects and T2DM patients for clinical research purposes, e.g. for testing control algorithms *in silico*.

Two test procedures in general use for the estimation of insulin sensitivity are the Intra-Venous Glucose Tolerance Test (IVGTT), which needs to be analyzed by means of a suitable mathematical model [9–11]; and the Euglycemic Hyperinsulinemic Clamp (EHC) [12], considered the ‘gold standard’ for the assessment of insulin resistance, which yields a measure of insulin sensitivity by direct averaging of the final glucose infusion rate. The standard IVGTT is simpler to perform than the EHC, has no significant associated risks and, if appropriately modelled, provides important information about the negative feedback regulation of glucose and insulin in a specific subject. Efforts have been made therefore to develop better models for the interpretation of the IVGTT experimental data set, exhibiting behaviour compatible with physiology (for example bounded and positive solutions of the model, as for example glucose predictions that never go to infinity or assume negative values) [13] and providing stable (assuming similar magnitude among different subjects), precise estimates of the structural model parameters [10]. Minimal models (minimal in the number of equations and in the number of parameters to be estimated) have therefore been developed to estimate the insulin sensitivity of a specific subject from a relatively non-invasive test procedure such the IVGTT. The so-called “Minimal Model” (MM) [9], which is still the most widely used compact model in the clinical setting, was demonstrated [11, 13] to suffer from a number of drawbacks, among which poor parameter estimation, with very large parameter coefficients of variation, translating into overall non-identifiability of the model parameters, including in particular the *S*_*I*_ index of insulin sensitivity. Moreover, the estimation procedure typically used, decoupling the feedback and estimating separately the two glucose-insulin control arms, provides misleading results as is discussed in greater detail elsewhere [10]. An alternative model of the compact model class, previously published by the present authors [10] and referred to as the Single Delay Model (SDM), was demonstrated to have mathematically consistent solutions, admitting the fasting state as its single equilibrium point and converging back to it from the perturbed state. This model was simultaneously fitted to glucose and insulin IVGTT observations on a heterogeneous population composed of lean, overweight, obese and morbidily obese subjects and was proven to outperform the MM over the whole range of conditions considered [11]. An extension of the SDM was subsequently proposed [14], where in order to model the fate of *per os* glucose, a gastrointestinal tract model was added to the plasma insulin and glucose dynamic equations and the glucose rate of appearance was derived by describing the absorption of glucose along a sequence of three gut compartments.

With the aim of interpreting a wide range of heterogeneous experimental results related to pancreatic insulin secretion, an islet population model was proposed in 2010 [15], incorporating the original idea of Grodsky of a population of independent controllers, coupled only by circulating glucose levels [16]. This model explains the effect of the islet response to varying glucose concentrations by means of a simple second-order nonlinear model, of the same functional form for all islets, with a random distribution of parameter values over the large number of islets considered. The population of pancreatic *β*-cells, collected into Langerhans islets, can be viewed therefore as a set of independent, similar, but not identical controllers (firing units) with distributed functional parameters. The islet equations are coupled to a metabolism sub-model to complete the description of the feed-back control of the glucose/insulin system dynamics. This islet population model was shown to reproduce very closely a wide array of actually observed, diverse *in vivo* and *in vitro* experiments, including the pioneering work of Grodsky, with the same set of working parameters [17]. While the model does not include any dependency on the rate of change of glycemia, it is able to reproduce accurately the double phase of insulin release during a prolonged glucose stimulus: a first phase of impulsive insulin release, immediately upon glucose administration, and a second phase of more gradual release, also linked with the potentiation effect of persistent hyperglycemia on the secretory units.

During the course of the development of the SDM in 2007 it was appreciated that, while the inclusion of some model components was irrelevant to the quality of data fitting (e.g. the inclusion of “glucose effectiveness” or an esplicit representation of the delay of insulin action on glucose), other elements, such as the delay *τ* and the exponent *γ*, were indispensable, and neglecting them led to the inability of the model to adequately fit the observations. Parameter *τ* represents the delay with which the pancreas changes secondary insulin release in response to varying glycemia, while the exponent *γ* represents the rapidity with which the insulin secretion rate reaches its maximum value with increasing glycemia. It was proven in [10] that both the delay *τ* and the exponent *γ* included into a nonlinear sigmoidal function were indeed necessary to interpret data.

Now, a few years after the publication of the SDM model, a comprehensive model for insulin secretion exists [15, 17], which is in fact able to explain mechanistically why a host of morphologically diverse insulin secretion responses to glycemic perturbations occur. We can therefore go back and attempt to understand why the nonlinearity *γ* was so essential in the representation of insulin secretion within the compact SDM model: this is the aim of the present work.

The reason why the islet population model is used as a comparison model is that it mirrors the actual anatomic structure of the endocrine pancreas and, at the same time, is able to convincingly reproduce observed insulin secretion patterns after many different types of glycemic perturbations.

Starting from the extended and validated model [17], the following procedure is implemented: a) a sample of IVGTT data sets is generated from the extended model portraying a group of virtual patients with a range of insulin sensitivities; b) the generated IVGTTs are fitted with the SDM compact model; c) a comparison is made between the set of generating extended model parameter values and the set of estimated compact-model parameter values to clarify their relationships.

## Methods

### Compact Model

**Fig 1:**
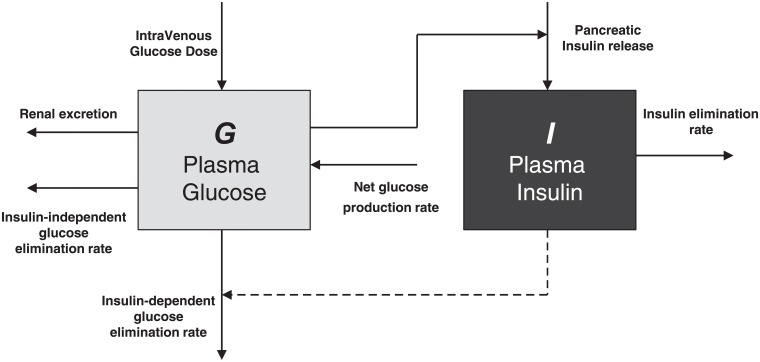
Schematic representation of the *Compact Model*. Glucose Plasma concentrations arise following an IntraVenous Glucose Dose. Input to plasma Glucose compartment comes also from the liver which produces glucose by means of gluconeogenesis and glycogenolysis processes, while elimination occurs by an independent (first order elimination rate) and dependent-insulin mechanism (second order tissue uptake). High plasma glucose concentrations enhance insulin release in plasma, while insulin is cleared with a first order elimination rate.

The delay model used in the present work, henceforth *Compact Model* (see Fig 1), has been shown to be able to represent well the glucose and insulin concentrations observed during an Intra-Venous Glucose Tolerance Test (IVGTT) [10]. Using this model it is possible to estimate the insulin sensitivity of a patient by fitting the patient’s IVGTT data, and this estimation is precise, reproducible and robust [11]. This model has been shown to admit mathematically consistent solutions with physiologically plausible parameter values [18].

Let *G*(*t*) and *I*(*t*) be, respectively, the plasma glucose concentration [mM] and the serum insulin concentration [pM], while their distribution volumes are indicated, respectively, as *V*_*G*_ [L/kgBW] and *V*_*I*_ [L/kgBW]. The differential equation representing variation of plasma glucose concentration is:

$$\frac{dG(t)}{dt}=-k_{XGI}I\left(t\right)G\left(t\right)+\frac{T_{gh}}{V_{G}},$$ (1)

with initial conditions:

$$\begin{array}{c} \begin{array}{cc} G(t) & =G_{b}\;\forall t\in \left(-\infty ,0\right),\;G\left(0\right)=G_{b}+G_{\Delta },\\ & \text{where}\;G_{\Delta }=\frac{D_{g}}{V_{G}}. \end{array} \end{array}$$ (2)

The glucose basal concentration *G*_*b*_ [mM] is the glycemia level before the bolus injection, while *G*_Δ_ [mM] represents glycemia increase following the bolus. *D*_*g*_ [mmol/kgBW] is the intravenous dose of glucose administered at time 0 during an IVGTT experiment. The equation representing the dynamics of plasma insulin concentration *I* [pM] is the following:

$$\frac{dI(t)}{dt}=-k_{XI}I\left(t\right)+\frac{T_{ig}^{\text{max}}}{V_{I}}\frac{{\left(\frac{G(t-\tau_{g})}{G^{*}}\right)}^{\gamma }}{1+{\left(\frac{G(t-\tau_{g})}{G^{*}}\right)}^{\gamma }},$$ (3)

with initial condition:

$$I\left(0\right)=I_{b}+I_{\Delta G}G_{\Delta },$$ (4)

The novelty this model introduced is in the second term of (3), representing second-phase insulin delivery from the *β*-cells. Its functional form is consistent with the hypothesis that insulin production is limited, reaching a maximal rate of release $T_{ig}^{\text{max}}/V_{I}$ by way of a Michaelis-Menten or a sigmoidal Hill dynamics according to whether the *γ* value is 1 or greater than 1 respectively. The constant values *T*_*gh*_ and $T_{ig}^{\text{max}}$ can be directly computed from the steady state conditions of Eqs (1) and (3) respectively. See the original references for more details [10, 11].

### Extended Model

The mathematical model of the glucose-insulin system presented in [17], henceforth *Extended Model*, has been the first model of whole-body insulin secretion to provide a unified explanation of an array of diverse clinical experimental procedures. In fact, the model is able to reproduce, with the same set of (meta)parameters: the low-frequency ultradian oscillations appreciable in the insulinemic signal when a constant enteral feeding is administered to a patient [19]; the entrainment of insulinemia to glycemia when a patient undergoes different Intra-Venous (I.V.) glucose administration patterns with different frequencies [20]; high-frequency insulinemia oscillations triggered by I.V. administration of very small amounts of glucose [21]; reproduction of the glycemia and two-stage-insulinemia (first and second phase) curves, upon simulated I.V. glucose administration during an IVGTT experiment. In this latter case, moreover, the model is able to mimic the clinically observable glycemia and insulinemia curves both for Normal Glucose Regulation (NGR) and for different pre-morbid and morbid conditions, such as Impaired Fasting Glycemia (IFG), Impaired Glucose Tolerance (IGT), IFG+IGT and Type 2 Diabetes Mellitus (T2DM).

While this model is extensively discussed elsewhere [17], we briefly summarize its features in the following.

**Fig 2:**
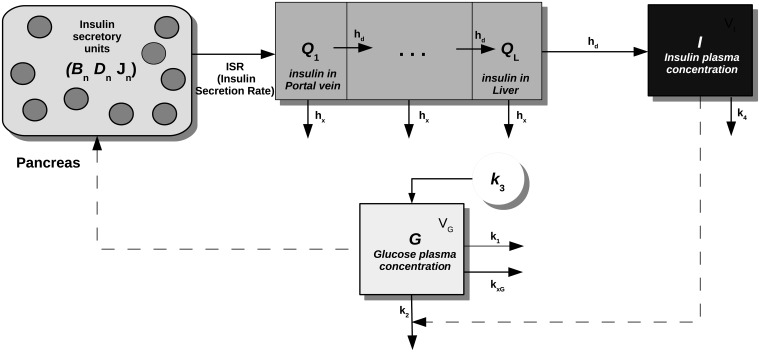
Schematic representation of the *Extended Model*. The pancreas secretory units (circles) release at different times their packet of insulin *J*_*n*_(*t*) (depending, for the *n*-th controller, on the threshold *B*_*n*_(*t*) and on the potentiation level *D*_*n*_(*t*)) in such a way that at any given time a total quantity *J*(*t*) (sum of the amounts *J*_*n*_(*t*)) of insulin flows into the portal vein (*Q*_1_ compartment), then to the liver (*Q*_*i*+1_…*Q*_*L*_ compartments) and finally reaching the plasma (compartment *I*), stimulating the uptake of glucose by tissues. Glycemia (*G* compartment), which is raised by glucose hepatic production *k*_3_, stimulates the production of insulin, closing the loop.

The basic paradigm of this model (see Fig 2) is that in the pancreas a multitude of similar, but not identical, independent controllers react to the sensed plasma glucose, which acts as the single “coupling” signal. While the qualitative behavior of the *firing units* is the same, each unit reacts differently to glycemia, and these heterogeneous performances yield the characteristic insulin responses to different stimuli. Note that the physiological identification of the firing unit could be the *β*-cells scattered in the pancreatic Langerhans islet, or, by choosing a different level of model granularity, subcellular granules, or, conversely, collections of synchronized *β*-cells within the islets of Langerhans (for more details, see [17]).

A firing unit (unit *n*) releases at time t* its stored packet of insulin *J*_*n*_(t*) [pmol/kgBW] whenever circulating glycemia exceeds the current threshold value *B*_*n*_(t*) [mM], thereupon entering a (relative) refractory state, where further stimulation fails to elicit the release of new hormone (the time-course of the threshold function *B*_*n*_(*t*) is in general different for each unit *n*); the refractory state of unit *n* is represented in the model by instantaneously increasing the glycemia threshold of that unit to a high level *R*_*n*_ [mM] whence, over time, it exponentially decreases towards its resting threshold value *G*_*n*_ [mM], *G*_*n*_ < *R*_*n*_. As soon as the controller fires, its threshold abruptly increases its value and the controller enters a refractory state. From this moment onwards, the threshold value decreases exponentially. When the threshold *B*_*n*_ reaches again values comparable with the current glycemia, the unit *n* is ready to release a new packet again. The size of the packet of insulin itself depends on prevailing glycemias: in fact, the well-known phenomenon of *potentiation* occurs, that is the ability of the pancreas to respond with progressively increasing insulin amounts to identical glucose stimuli, when these are repeated in close proximity over time [16, 22, 23]. The variation in size of the packet of insulin (subject to potentiation) for each firing unit is described by the equation for *D*_*n*_(*t*) [pmol/kgBW] [17].

Each different firing unit *n* is then characterized by the same triple of equations (*B*_*n*_(*t*), *D*_*n*_(*t*), *J*_*n*_(*t*)), with different parameter values. As described in depth elsewhere [17], their values are randomly sampled from given (usually lognormal) distributions.

The differential equation associated to *B*_*n*_ is:

$$\frac{dB_{n}\left(t\right)}{dt}=-\alpha_{n}B_{n}\left(t\right)+\alpha_{n}G_{n}+\left(R_{n}-B_{n}\left(t\right)\right)\delta (\chi (\left\{G\left(t\right)<B_{n}\left(t\right)\right\})),$$ (5)

where *α*_*n*_ [min^−1^] is the rate of recovery of sensitivity of the secretory unit, *G*(*t*) [mM] is the external glycemia sensed by all the secretory units (that is the glucose plasma concentration), and *δ*(⋅) is a Dirac delta term specifying instantaneous increase of the threshold to the refractory level *R*_*n*_, associated with discharge of insulin, at any time the glucose stimulus *G*(*t*) exceeds the controller threshold *B*_*n*_; *χ* is the characteristic function of its argument set.

The parameter *G*_*n*_ is the only one that is not extracted from a lognormal distribution, but instead from a distribution with the following density (see for more details [17]):

$$f\left(g\right)=\nu g_{1/2}^{\nu }\frac{g^{\nu -1}}{{\left(g^{\nu }+g_{1/2}^{\nu }\right)}^{2}},$$ (6)

depending on the two parameters *g*_1/2_ [mM] and *ν* [#].

The *Insulin Secretion Rate* (ISR) is defined as:

$$\text{ISR}\left(t\right)=\sum_{n=1}^{N}J_{n}\left(t\right)\delta (\chi (\left\{G\left(t\right)<B_{n}\left(t\right)\right\})),$$ (7)

where *N* is the total number of firing units. Basically, Eq (7) states that the ISR at time *t* is given by the sum of the insulin packets fired by the units whose threshold *B*_*n*_(*t*) is smaller than or equal to the current glycemia *G*(*t*). Insulin then flows to the portal vein and the liver according to:

$$\frac{dQ(t)}{dt}=-h_{x}Q\left(t\right)+\text{ISR}\left(t\right)$$ (8)

where *Q* [pmol/kgBW] refers to the insulin amount in the portal vein/liver compartment. Then, insulin mass *Q* enters the plasma insulin distribution space according to the following equation:

$$\frac{dI(t)}{dt}=-k_{4}I\left(t\right)+\frac{h_{d}Q\left(t\right)}{V_{I}},$$ (9)

where *I*(*t*) [pM] is the serum insulin concentration. Finally, glucose plasma concentration is described by:

$$\frac{dG(t)}{dt}=-k_{1}\tilde{u}\left(G\left(t\right)\right)-k_{2}I\left(t\right)G\left(t\right)+\frac{k_{3}\left(t\right)}{V_{G}}.$$ (10)

where:

1. the first term in (10) describes approximately the (supra-threshold) driving glycemia for urinary glucose elimination:
  $$\tilde{u}\left(G\right)=\{\begin{array}{cc} 0, & G<G_{u},\\ G-G_{u}, & G\geq G_{u}. \end{array}$$ (11)
  with *k*_1_ [/min] the apparent insulin-independent renal elimination rate for glucose, occurring at glycemias greater than the threshold *G*_*u*_ [mM];
2. the second term is the insulin-dependent glucose uptake, with *k*_2_ [/min/pM] being the rate of glucose uptake by tissues per pM of serum insulin concentration;
3. the third term *k*_3_(*t*) [mmol/kgBW/min] refers to the net balance between hepatic glucose output and insulin-independent zero-order glucose tissue uptake (essentially by the brain), with *V*_*G*_ [L/kgBW] the apparent distribution volume for glucose.

In order to account for the noisy time-course of the last term *k*_3_, a stochastic model has been employed:

$$k_{3}\left(t\right)={\bar{k}}_{3}+\tilde{s}\left(\xi \left(t\right)\right),$$ (12)

where ${\bar{k}}_{3}$ [mmol/kgBW/min] is a central value for *k*_3_(*t*) and *ξ*(*t*) [mmol/kgBW/min] is a stochastic process. For further details, see [17].

### Modifications of the two models

In the present work, we use the fact that the *Compact Model* [10] is able to fit IVGTT data of a virtual patient generated by the *extended model* [17]. In a previous work [24] we obtained very good fits of the Compact Model on the Extended Model using directly parameters reported in [17] for generating virtual patients with profiles ranging from NGR to T2DM; nevertheless, we introduce here some modifications of the Extended Model in order to better reproduce observed IVGTTs from a larger sample of clinical tests.

In particular, since both the *Extended* and the *Compact Model* do not present any term of insulin-independent glucose elimination, we modified the glycemia Eqs (1) and (10) respectively as:

$$\frac{dG(t)}{dt}=-k_{XGI}I\left(t\right)G\left(t\right)+\frac{T_{gh}}{V_{g}}-k_{XG}G\left(t\right)$$ (13)

and

$$\frac{dG(t)}{dt}=-k_{1}\tilde{u}\left(G\left(t\right)\right)-k_{2}I\left(t\right)G\left(t\right)+\frac{k_{3}\left(t\right)}{V_{G}}-k_{XG}G\left(t\right),$$ (14)

Consequently from (13), the new value for *T*_*gh*_ is computed at the equilibrium as:

$$T_{gh}=\left(k_{XGI}I_{b}G_{b}+k_{XG}G_{b}\right)V_{G}.$$ (15)

It is to be noted that the Compact Model does not include the glycosuria term, which is instead present in the Extended Model—see Eq (10).

### Virtual patient fitting

**Fig 3:**
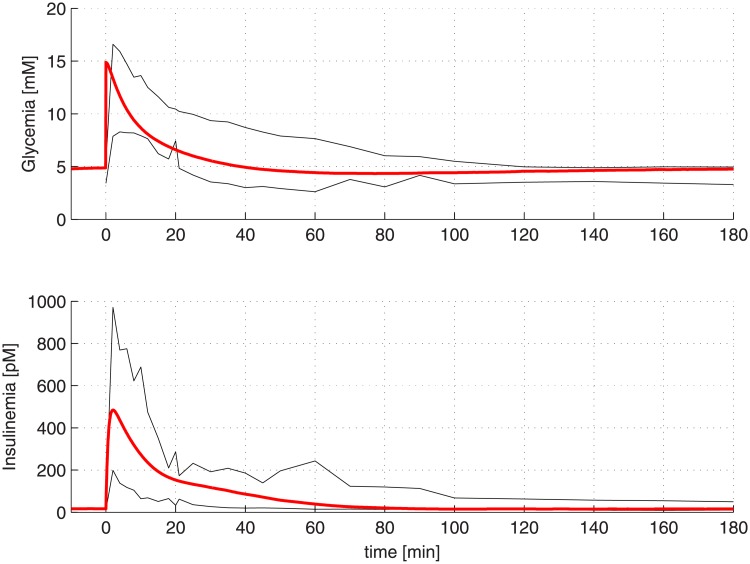
Median of 100 virtual NGR patients generated by the Extended Model (red curves). The black curves represent the 5th and 95th percentile of the data analyzed in [10].

Before producing virtual patients with the Extended Model, some fine tuning of the model parameters was thought to be advisable in order to better match glycemia and insulinemia trends of Normal Glucose Regulation (NGR) patients undergoing an Intra-Venous Glucose Tolerance Test (IVGTT), as reported in the literature (original data which served to set the best parameters values derived from Panunzi *et al.* [10], from which only the subsample of lean subjects was considered). A few modifications in the parameter/metaparameter values of the Extended Model have therefore been carried out, as reported in Table 1: as it may be noticed, the glucose net balance mean value (${\bar{k}}_{3}$) has been halved, while the mean value of the rate of recovery of sensitivity of the secretory unit (*α*_*n*_) has been decreased, obtaining a slower insulin dynamics. Moreover, the renal glucose elimination threshold *G*_*u*_ has been increased according to the literature. In Fig 3 are reported as thin black lines the 5th and 95th percentile of the data analyzed in [10], while the median of 100 virtual NGR patients generated by the Extended Model with the new parameter values reported in Table 1 is shown as a thick red line. This trend is coherent with NGR glycemia and insulinemia curves observed during IVGTTs as reported in the literature [25–27].

**Table 1:** Changed parameter values of the Extended Model with respect to [17].

Symbol	Units	Mean	Std. deviation	Value
*α*_*n*_	min^−1^	7.5 × 10^−2^	0.12	–
${\bar{k}}_{3}$	mmol/kgBW/min	–	–	5 × 10^−3^
*G*_*u*_	mM	–	–	11
*k*_*XG*_	min^−1^	–	–	3 × 10^−3^

With this new set of (meta-)parameters, virtual patients exhibiting different degrees of disease (ranging from a Normal Glucose Regulation (NGR) patient to a Type-2 Diabetes Mellitus (T2DM) patient) were generated by modifying parameters as reported in Table 2. In the same way as previously done [17], we obtained virtual T2DM patients by lowering peripheral insulin-sensitivity (parameter *k*_2_), increasing liver glucose production ${\bar{k}}_{3}$ (reflecting lower hepatic insulin sensitivity), by lowering the size of the insulin packets (parameters *μ*(*ρ*_*n*_) and $\mu ({\bar{D}}_{n})$), and by lowering firing thresholds (whose distribution depends on the parameter *g*_1/2_). The resulting pancreatic behavior still shows a higher insulin production given by the higher number of recruited firing units (due to lower firing thresholds), in the effort to compensate the lower insulin sensitivity and under the hypothesis that these T2DM patients are still able to compensate. However, the ability of these subjects to further increase their pancreatic insulin secretion responding to higher glycemias is severely limited.

**Table 2:** *Compact Model* parameter estimates for a NGR, pre-diabetic and T2DM virtual patient (see Figs 4, 5 and 6).

Par.	NGR	Pre-diabetic	T2DM
*I*_Δ_	49.8	38.592	31.03
*τ*_*g*_	19.65	18.85	20.275
*k*_*XGI*_	1.19 × 10^−4^	7.04 × 10^−5^	3.66 × 10^−5^
*k*_*XI*_	7 × 10^−2^	7.4 × 10^−2^	5.5 × 10^−2^
*γ*	3.6473	5.2394	6.687

We generated IVGTT glycemia and insulinemia curves for a total of 500 virtual patients, divided into 25 degrees of morbidity, ranging from “pure” NGR to “pure” T2DM condition in 25 uniform steps (see Table 3); this choice is a good tradeoff between the computational time required to generate all the cohort and the value necessary to have an “average” patient for each class. For each degree of morbidity, 20 virtual patients were generated exhibiting somewhat different behavior due to the stochasticity intrinsic in the Extended Model (given by the randomly generated structural parameters). Glycemia and insulinemia curves of T2DM patients undergoing IVGTTs as reported in the literature [27–29] are comparable to the ones generated by the Extended Model.

**Table 3:** Parameters varying values for T2DM patient undergone an IVGTT.

Par.	NGR	T2DM
*k*_2_	1.4 × 10^−4^	0.1 × 10^−4^
${\bar{k}}_{3}$	0.005	0.005 × 1.6
*μ*(*ρ*_*n*_)	6.5 × 10^−3^	6.5 × 10^−3^ ⋅ 0.05
$\mu ({\bar{D}}_{n})$	3 × 10^−3^	3 × 10^−3^ ⋅ 0.3
*g*_1/2_	9.7697	4

Observation data were then extracted for each virtual patient from the glycemia and insulinemia curves at times −10, −5, −0.5, 2, 4, 6, 8, 10, 15, 20, 25, 30, 40, 50, 60, 90, 120 min. The “observations” were finally fitted with the Compact Model, estimating the following vector of parameters ***θ***:

$$\begin{array}{c} \theta ={\left(\begin{array}{ccccc} I_{\Delta } & \tau_{g} & k_{XGI} & k_{XI} & \gamma \end{array}\right)}^{T}. \end{array}$$ (16)

Since the simulated glucose bolus has been set in such a way to produce a sudden glycemia increment of 10 mM for every patient, *G*_Δ_ in Eq (2) was fixed to 10 mM as well. The glucose distribution volume *V*_*G*_ was set to 0.2 L/kgBW and kept fixed during the generation of all virtual patients.

The fit for each virtual patient was performed by solving a constrained optimization problem (lower and upper bounds were considered) using a Nelder-Mead algorithm minimizing the following loss function λ:

$$\lambda ={\left(\frac{G_{i}-{\hat{G}}_{i}}{{\hat{G}}_{i}}\right)}^{2}+{\left(\frac{I_{i}-{\hat{I}}_{i}}{{\hat{I}}_{i}}\right)}^{2},$$ (17)

where *G*_*i*_ and *I*_*i*_ are observed glycemia and insulinemia at time *t* = *i*, whereas ${\hat{G}}_{i}$ and ${\hat{I}}_{i}$ are the corresponding estimated glycemia and insulinemia. This function was adopted in order to consider at the same time both glycemia and insulinemia data, using a normalization factor.

## Results

**Fig 4:**
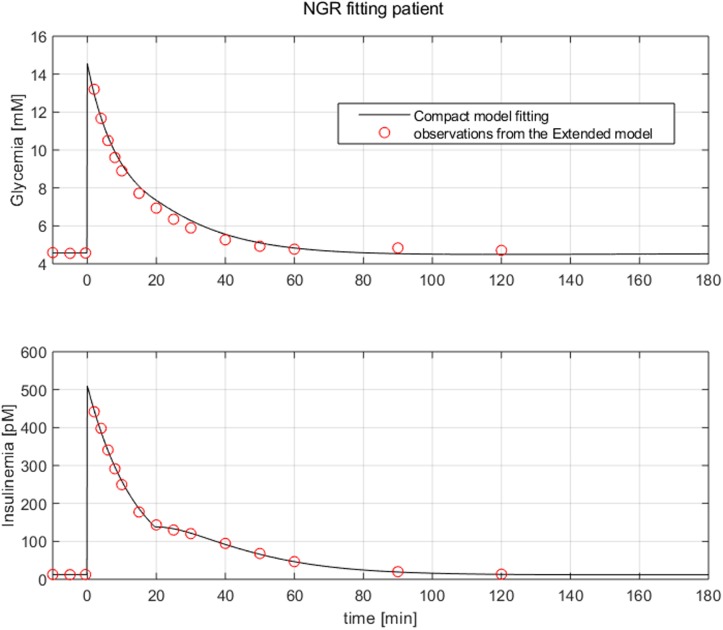
Observations extracted from a virtual NGR patient undergone an IVGTT (red dots). The black line represents the Compact Model fitting.

**Fig 5:**
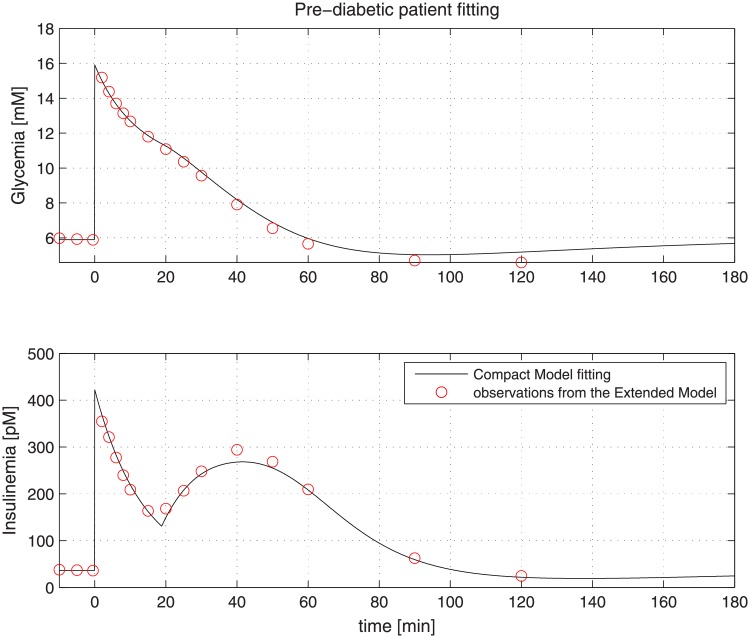
Observations extracted from a virtual prediabetic patient undergone an IVGTT (red dots). The black line represents the Compact Model fitting.

**Fig 6:**
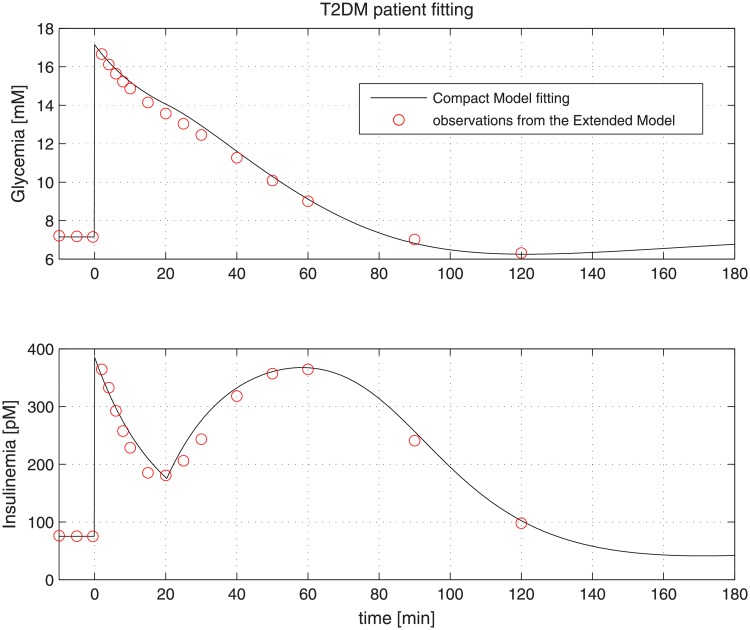
Observations extracted from a virtual T2DM patient undergone an IVGTT (red dots). The black line represents the Compact Model fitting.

Virtual NGR patients were generated by using the same model parameter values employed for producing curves reported in Fig 3. Fig 4 reports the result of the fitting procedure on data extracted from a virtual NGR patient, while Figs 5 and 6 report the fitting performance for a virtual pre-diabetic patient and for a virtual T2DM patient respectively; Table 2 reports the Compact Model estimated parameters compared with the generating Extended Model parameter values.

**Fig 7:**
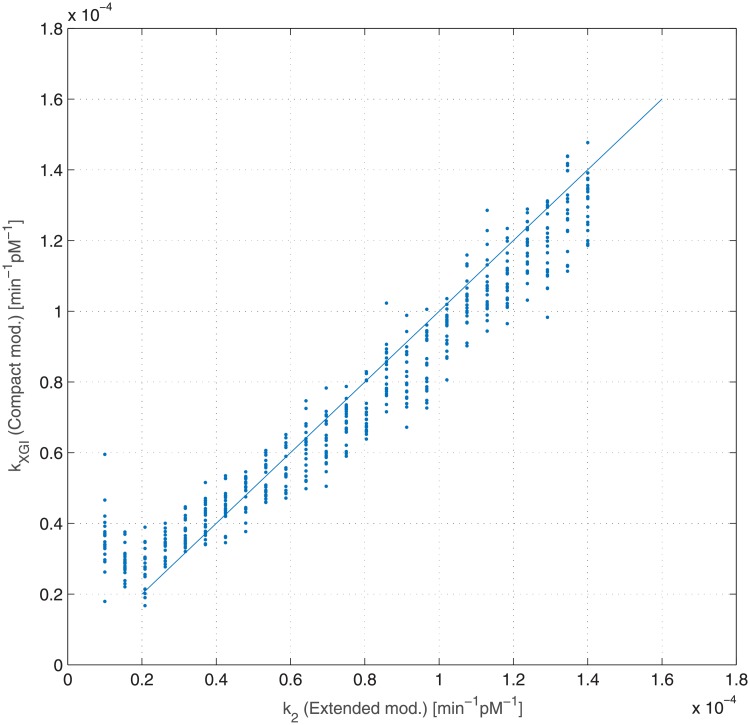
Dependence of estimated insulin sensitivity *k*_*XGI*_ (Compact Model) from generating insulin sensitivity *k*_2_ (Extended Model). Each dot represents a single virtual patient, while the line is the bisector *k*_*XGI*_ = *k*_2_. Coefficient of correlation and its associated P-value: *r* = 0.97 (*P* < 0.001).

Since the insulin-sensitivity parameter is present in both models (*k*_*XGI*_ for the Compact Model and *k*_2_ for the Extended Model), we reported in the same plot (Fig 7) the relation between the generating *k*_2_ parameter values and the corresponding estimated *k*_*XGI*_ values: as it is clear from the figure, the cloud of points lies mostly along the line *k*_*XGI*_ = *k*_2_, showing that the Compact Model [10] and the Extended Model are very consistent in particular for what concerns insulin sensitivity.

**Fig 8:**
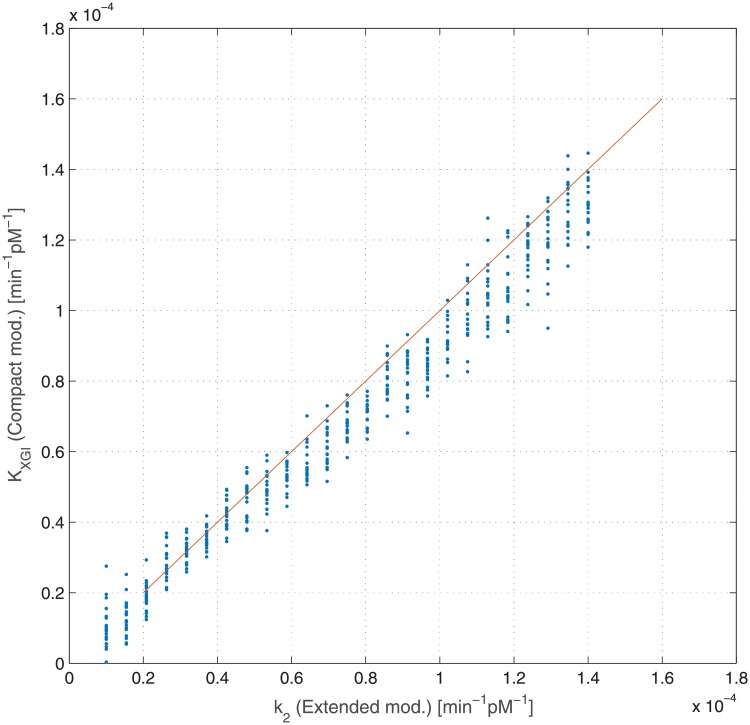
Dependence of estimated insulin sensitivity *k*_*XGI*_ (Compact Model, with glycosuria term) from generating insulin sensitivity *k*_2_ (Extended Model). Each dot represents a single virtual patient, while the line is the bisector *k*_*XGI*_ = *k*_2_. Coefficient of correlation and its associated P-value: *r* = 0.98 (*P* < 0.001).

Fig 8 reports the same relationship when renal glucose output was added to the Compact Model. In this case the points lie wholly on the identity line.

**Fig 9:**
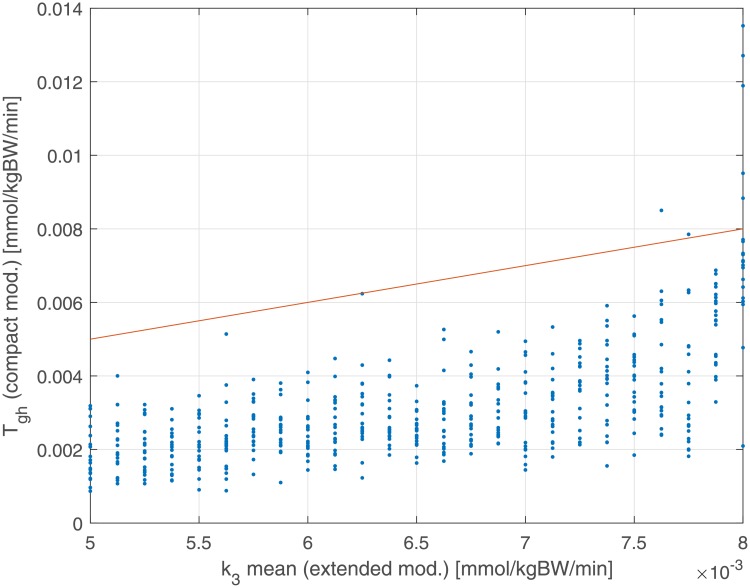
Dependence of estimated glucose net balance *T*_*gh*_ from the generating ${\bar{k}}_{3}$ parameter. The red line represents the bisector $T_{gh}={\bar{k}}_{3}$. Coefficient of correlation and its associated P-value: *r* = 0.62 (*P* < 0.001).

Fig 9 shows the relationship between generating average HGO from the Extended Model (${\bar{k}}_{3}$) and the corresponding *T*_*gh*_ parameter from the Compact Model. As can be seen there is a systematic underestimation of net HGO by the Compact Model.

**Fig 10:**
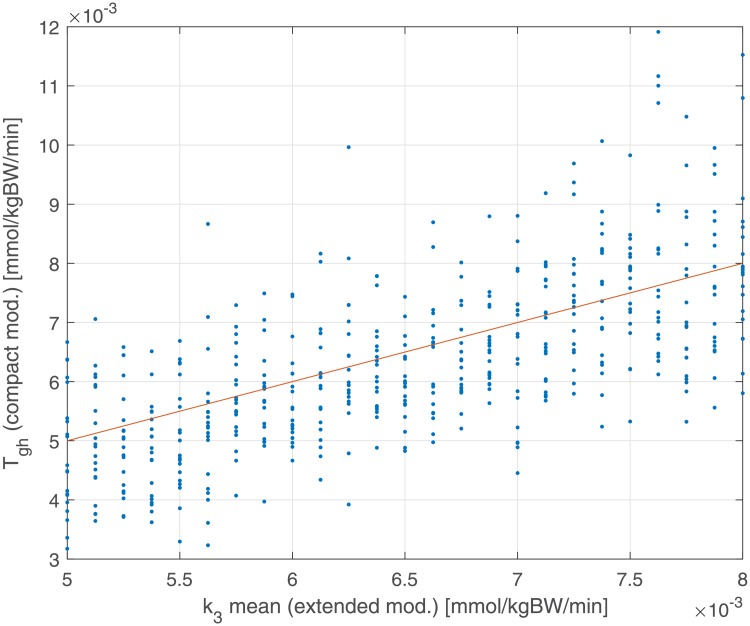
Dependence of estimated glucose net balance *T*_*gh*_ (from the Compact Model comprehensive of the glycosuria term) from the generating ${\bar{k}}_{3}$ parameter. The red line represents the bisector $T_{gh}^{\prime }={\bar{k}}_{3}$. Coefficient of correlation and its associated P-value: *r* = 0.68 (*P* < 0.001).

In Fig 10 we report in the same plot (estimated net HGO *T*_*gh*_ against the generating net HGO ${\bar{k}}_{3}$) when the Compact Model includes the glycosuria term. In Fig 10 we notice that the cloud of points lies around the line $T_{gh}={\bar{k}}_{3}$: it is clear that the underestimation by the original version of the Compact Model depends on having neglected glycosuria.

We recall that the relationship between circulating glycemia and insulin secretion for each virtual patient depends on the threshold distribution of the parameters *G*_*n*_ (see Eq (5)), randomly extracted from the distribution *f*(*g*) in Eq (6). The shape of this function depends on the pathophysiological condition of the virtual patient and therefore on the value of its *g*_1/2_ parameter (see Table 3).

We now define *z* ∈ [0, 1] [#] to be a coefficient of *pancreatic reserve*, representing, for each given subject, the fraction of total firing units able to react to an abrupt raising in glycemia above the baseline. It can be computed as:

$$z=1-\int_{0}^{G_{b}}\;\;\;f\left(g\right)dg=1-\frac{G_{b}^{\nu }}{G_{b}^{\nu }+g_{1/2}^{\nu }}=\frac{g_{1/2}^{\nu }}{G_{b}^{\nu }+g_{1/2}^{\nu }},$$ (18)

where *G*_*b*_ [mM] is the basal glycemia of the patient (before the injection of the glucose bolus), *ν* = 2.5137 [#] (see [17]) is constant for all patients and *g*_1/2_ [mM] varies, passing from NGR to T2DM, according to Table 3.

**Fig 11:**
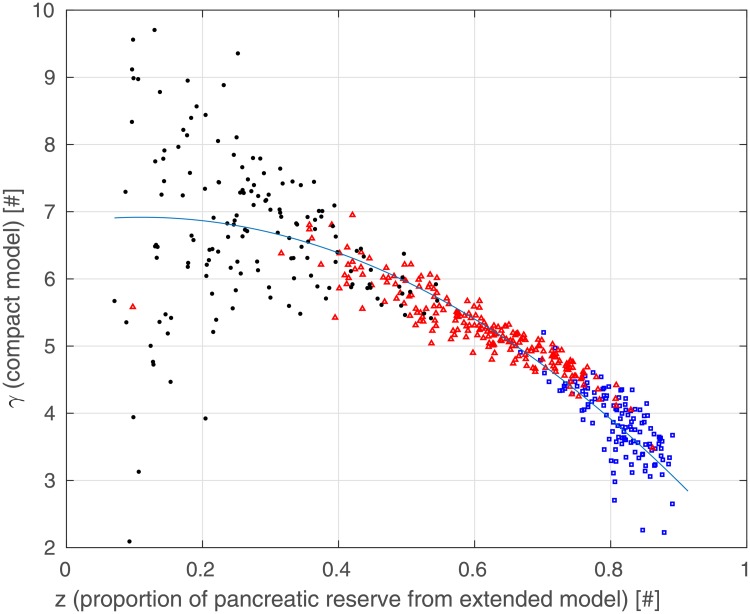
Dependence of estimated Compact Model parameter *γ* from proportion of pancreatic reserve *z* (extracted from generating Extended Model data) for NGR (blue squares), pre-diabetic (red triangles) and T2DM (black dots) patients.

Fig 11 shows the relationship between pancreatic reserve *z* and estimated parameter *γ* from Eq (3), for each simulated subject. The virtual patients portrayed in the figure are identified with a different colored marker depending on their estimated insulin sensitivity ${\hat{k}}_{XGI}$ [min^−1^pM^−1^]: *healthy* (${\hat{k}}_{XGI}\geq 1\times 10^{-4}$, blue square), *pre-diabetic* ($0.5\times 10^{-4}\leq {\hat{k}}_{XGI}<1\times 10^{-4}$, red triangle) and *diabetic* (${\hat{k}}_{XGI}<0.5\times 10^{-4}$, black dot). In this figure, healthy and pre-diabetic patients cluster into two tight distinct sets; diabetic patients are scattered over a different, widely diffused set. The three sets present a clear differentiation based on the value of the pancreatic reserve *z*: healthy subjects exhibit a high pancreatic reserve ranging from 0.7 to 0.9, pre-diabetic patients are characterized by a lower reserve value ranging from 0.4 to 0.8 and diabetic subjects present with a very low index of pancreatic reserve, below 0.4. NGR and pre-diabetic patients exhibit estimated values of *γ* which lie in the neighborhood of a falling parabola on the plane *z*-*γ*, reported in Fig 11 as a blue curve, their *γ* increasing as the pancreatic reserve *z* decreases. The diabetic patients, instead, although having low values of *z*, exhibit widely different estimated values for *γ*. The behavior of the *z*-*γ* relationship for normal and prediabetic subjects is clear. In the case of diabetic patients, the actual pancreatic reserve is so low, and the maximal possible increase in insulin secretion is so small, that the *γ* parameter from the Compact Model is difficult to identify precisely, with the corresponding worsening of the scatter of the estimates as *z* goes to lower and lower values.

**Fig 12:**
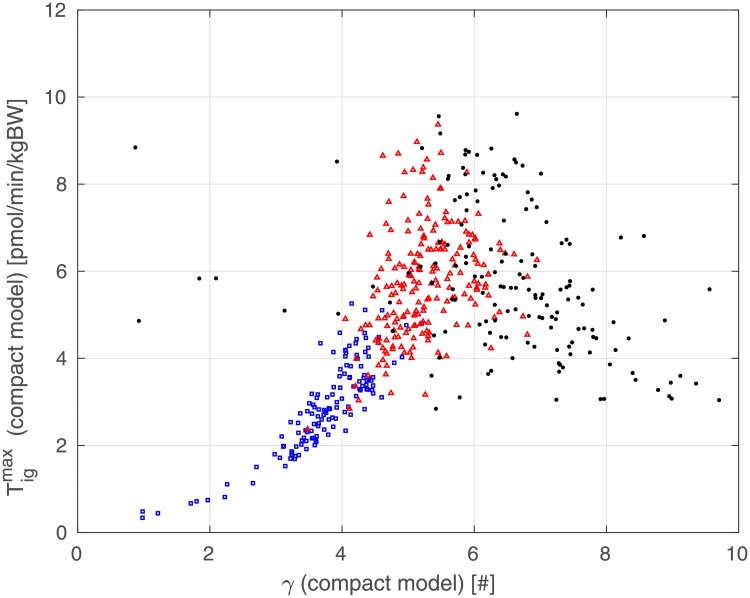
Dependence of Compact Model parameter $T_{ig}^{\text{max}}$ (determined from estimated parameters) from estimated Compact Model parameter *γ* for NGR (blue squares), pre-diabetic (red triangles) and T2DM (black dots) patients.

Fig 12 shows, for the 500 virtual patients, the relationship between the estimated *γ* and the value of $T_{ig}^{\text{max}}$ for the Compact Model, representing the maximum reachable Insulin Secretion Rate. The value $T_{ig}^{\text{max}}$ has been computed as follows:

$$T_{ig}^{\text{max}}={\hat{K}}_{xi}I_{b}V_{i}\frac{1+{\left(\frac{G_{b}}{G^{*}}\right)}^{\gamma }}{{\left(\frac{G_{b}}{G^{*}}\right)}^{\gamma }},$$ (19)

where ${\hat{K}}_{xi}$ [min^−1^] is the estimated constant disappearance rate for insulin, *I*_*b*_ is the basal insulinemia of the patient, *V*_*I*_ = 0.25 [L⋅kgBW^−1^] is the constant distribution volume for the virtual patient (from the *Extended Model*) and G* = 9 [mM] is the glycemia at which the insulin secretion rate is half of its maximum and is fixed for all patients. Both *V*_*I*_ and G* values are the same as in [10].

Also for Fig 11, when plotting $T_{ig}^{\text{max}}$ against *γ*, the subjects cluster again in three well defined groups: NGR patients (blue squares) exhibiting low values of *γ*, present a $T_{ig}^{\text{max}}$ which is typically below 4 pmol/min/kgBW; pre-diabetic patients (red triangles) exhibit higher values of *γ* and higher $T_{ig}^{\text{max}}$; diabetic patients (black dots), showing values of *γ* in a wide range (due to the above-mentioned difficulty of precisely estimating *γ* when increments of insulin secretion are limited), present a $T_{ig}^{\text{max}}$ above 3 [pmol/min/kgBW]. This pattern is consistent with the modest needs for increased insulin secretion in healthy, insulin-sensitive subjects, in whom, on the other hand, no ceiling for insulin secretion is readily apparent, hence the low nonlinearity coefficient *γ*, and vice versa for insulin-resistant subjects.

**Fig 13:**
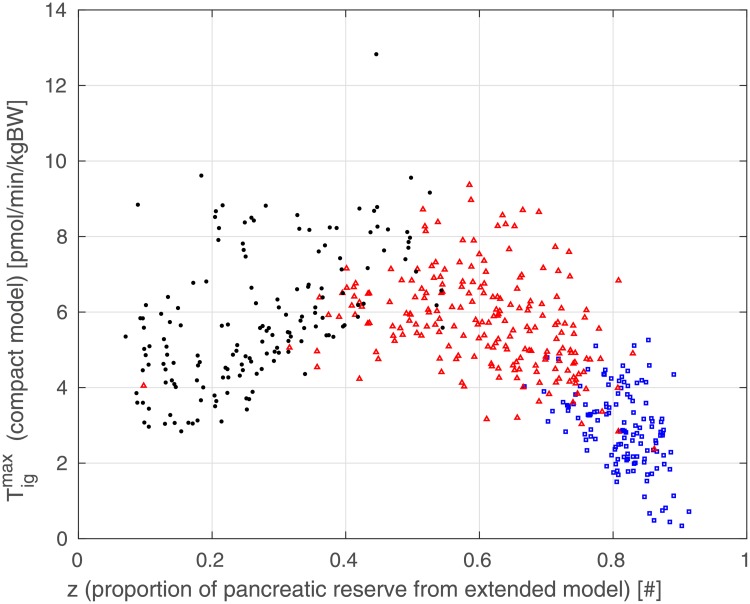
Dependence of Compact Model parameter $T_{ig}^{\text{max}}$ (determined from estimated parameters) from proportion of pancreatic reserve *z* (extracted from generating Extended Model data) for NGR (blue squares), pre-diabetic (red triangles) and T2DM (black dots) patients.

Fig 13 reports the dependency of $T_{ig}^{\text{max}}$ on *z* for each patient. We may observe the same clear separation among the three different classes of patients apparent in the other plots.

It can be seen that NGR patients exhibit a high value of pancreatic reserve *z* and low values of $T_{ig}^{\text{max}}$, since they do not need high insulin production due to their good insulin sensitivity; conversely, pre-diabetic and diabetic patients, with a lower pancreatic reserve, have a higher $T_{ig}^{\text{max}}$, since they need to compensate their lower insulin-sensitivity.

## Discussion

In the past 50-60 years many attempts at mathematical modelling of different aspects of the glucose-insulin system have been made. Some contributions concentrated more on the insulin secretion mechanisms [15, 16, 30], others more with short-term modelling of perturbation experiments [9, 10, 13, 14, 31], and some publications describe more complex models, from multi-organ models [8, 32] to maximal models for *in-silico* Type 1 diabetes virtual patient simulation [33–37].

Our group has been active in this area for many years. We have addressed in particular total body or organ-level integrated mechanisms, modelling both short-term experiments [10, 11, 13, 14, 18], pancreatic insulin release mechanisms based on firing units [15, 17, 24], long term compensation and disease development [38], incretin effect [39].

Our overall goal is to eventually obtain a coherent suite of models addressing different relevant clinical issues (e.g. IVGTT and OGTT for insulin sensitivity determination, or dynamics of insulin secretion), suitable for integration into an overall scheme. These models should be individually effective in dealing with the specific dynamics they approximate, should exhibit appropriate qualitative behavior from a mathematical viewpoint, should have desirable statistical properties leading to *a-posteriori* parameter identifiability and should also be consistent with one another, shedding light on the actual mechanisms involved in glucose homeostasis.

In particular, the clinical use of a compact, identifiable model should be justified by our confidence that it does capture the basic relevant physiology, if not all possible features of interest. In the present work we are trying to establish the relationship between an extended, validated model (which by its very size cannot be fruitfully employed for the analysis of limited experimental data e.g. from an IVGTT) and a Compact Model of proven effectiveness in estimating insulin sensitivity from small datasets. By generating virtual patients from the Extended Model and fitting them with the Compact Model we are able to link variations in the (simulated) physiology with diagnostic parameter value changes.

Some modifications of the Extended Model parameter values (with respect to previously published values [17]) were found to be necessary in order for simulated IVGTT curves to lie squarely within 90% confidence bounds obtained from sets of clinically recorded IVGTT’s from NGR subjects. These minor changes in the parameter values, however, did not affect the reproducibility of the diverse patterns generated from the whole array of experimental procedures, such as the slow (ultradian) [19] and fast oscillations [21], and the entrainment phenomenon due to sinusoidal glucose infusions [40], which were all re-checked with the modified parameter set.

Moreover, in order to align the two models, an insulin-independent glucose elimination term was introduced in the glycemia equation of the Extended Model. The insulin secretion formalization remains, of course, radically different between the complex Extended and the simple Compact Models.

Data of 19 patients from previous clinical studies [11] were used to build 90% confidence envelopes of the observed glycemia and insulinemia time courses.

Fig 3 shows that for the set of chosen parameters the median of 100 virtual NGR patients undergoing an IVGTT experiment lies within the 90% envelopes from real observations highlighting the ability of the Extended Model to reproduce well real IVGTT data.

Starting from the NGR parameter values, progressive degrees of clinical worsening (up to T2DM) were simulated by changing some model parameters, such as insulin sensitivity (*k*_2_), average net hepatic glucose production (${\bar{k}}_{3}$), and other parameters related to the mechanisms of insulin production (average *ρ*_*n*_, average ${\bar{D}}_{n}$, and *g*_1/2_).

The Compact Model was then fitted onto the IVGTT data generated with the Extended Model, and some of the Compact Model estimated parameters were compared with the corresponding values of the generating parameters.

Figs 4 to 6 clearly show the good performance of the Compact Model to fit the Extended Model curves, highlighting its ability to reproduce both first and second phases of insulin release. It is evident that as healthy conditions are altered to pre-diabetic and then to diabetic status, the second phase of insulin release appears to be more and more prominent, probably in order to compensate a higher insulin resistance translating into a lesser degree of glucose uptake and persistently high plasma glucose concentrations.

The consistence and robustness of the Compact Model is evident from Fig 7 where the relationship between the two model parameters *k*_2_ (for the Extended Model) and *k*_*XGI*_ (for the Compact Model), both quantifying insulin sensitivity, approximates very well the identity line in the *k*_2_-*k*_*XGI*_ plane.

The overestimation of the insulin sensitivity index (*k*_2_) for very high insulin resistance patients is due to the absence, in the Compact Model, of the term representing glucose renal elimination, which is instead present in the Extended Model. While, in principle, this structural difference could be absorbed by a lower net HGO (*T*_*gh*_), this compensation mechanism is insufficient (Fig 9) as it is glucose-independent: this causes a higher glucose uptake, which in the model is represented by a second order term (in glycemia and in insulinemia). Figs 8 and 10, compared with the corresponding Figs 7 and 9, show how the introduction of the glycosuria term in the Compact Model restores the linear relationship between *k*_*XGI*_ and *k*_2_ even at low insulin sensitivities, as well as solving the underestimation problem of *T*_*gh*_ with respect to ${\bar{k}}_{3}$ (the net HGO in the Extended Model).

It is reassuring, while not surprising, that variations in insulin sensitivity (from 0.1 × 10^−4^ to 1.4 × 10^−4^) in the generating Extended Model are reliably captured by the Compact diagnostic model. This supports the use of the Compact SDM model in clinical practice, lending credibility to the measures obtained from it.

Of greater interest is however the observation portrayed in Fig 11. When pancreatic reserve is maintained (*z* > 0.4, see Eq (18)), there is a clear (inverse) relationship between said pancreatic reserve and the nonlinear behavior of the pancreatic insulin release represented by parameter *γ*. When pancreatic reserve is exhausted, the increase in insulin secretion is too small with respect to the increase in glycemia with resulting very variable values of the nonlinearity exponent *γ*. Fig 13 shows that the maximum insulin production $T_{ig}^{\text{max}}$ is small in athletes or very healthy subjects (the pancreas in these subjects is not typically stimulated very much since insulin sensitivity is excellent), increases with BMI and prediabetes (consistently with clinical observations of high insulinemias in these subjects) and then decreases as the clinical picture worsens and (relative) pancreatic insufficiency develops; again consistently with clinical observations.

## Conclusion

A refinement of our understanding of the population-of-controllers interpretation of the insulin secretion behavior of the pancreas (in the several degrees of impairment between NGR and T2DM) explains why the Compact Model for the IVGTT needs to explicitly incorporate the saturating nonlinearity of insulin secretion with increasing glycemia, and the fact that this nonlinearity worsens with progressive worsening of the clinical picture.

This consistency in the behavior of the two models, developed for and fitted on very different sets of data, increases our confidence that both models, each in its own domain, are reliable representations of the actual physiology.

It is remarkable that the Compact Model, with only two parameters relative to the insulin secretion mechanism, and estimable on the small datasets from IVGTT experiments, is able to capture a series of detailed physiological mechanisms explicated and represented in the Extended Model: the end-result of a very complex set of events, such as the potentiation and synchronization of the whole *β*-cell population, along with the introduction of a heterogeneous behaviour of the secretory units (from the distribution of thresholds to the distribution of the rate of recovery of sensitivity) appears to be well captured by the very simple but specific nonlinear function of the Compact Model.

## References

[1] Bolie VW. Coefficients of Normal Blood Glucose Regulation. Journal of Applied Physiology. 1961;16:783–788. 10.1152/jappl.1961.16.5.783 13870789

[2] Cobelli C, Kovatchev B. Artifical pancreas: past, present, future. Diabetes. 2011;60:2672–2682. 10.2337/db11-0654 22025773 PMC3198099

[3] Hovorka R. Closed-loop insulin delivery: from bench to clinical practice. Nature Reviews Endocrinology. 2011;7(7):385–395. 10.1038/nrendo.2011.32 21343892

[4] Kovacs L, Szalay P, Almassy Z, Barkai L. Applicability Results of a Nonlinear Model-Based Robust Blood Glucose Control Algorithm. Journal of Diabetes Science and Technology. 2013;7(3):708–716. 10.1177/193229681300700316 23759404 PMC3869139

[5] Palumbo P, Pizzichelli G, Panunzi S, Pepe P, De Gaetano A. Model-based control of plasma glycemia: test on populations of virtual patients. Matematical Biosciences. 2014;257:2–10. 10.1016/j.mbs.2014.09.003 25223234

[6] Marchetti L, Reali F, Dauriz M, Brangani C, Boselli L, Ceradini G, et al A Novel Insulin/Glucose Model after a Mixed-Meal Test in Patients with Type 1 Diabetes on Insulin Pump Therapy. Scientific Reports. 2016;6 10.1038/srep36029 PMC5099899 27824066

[7] Kovatchev BP, Breton M, Dalla Man C, Cobelli C. In silico preclinical trials: a proof of concept in closed-loop control of type 1 diabetes. Journal of Diabetes Science and Technology. 2009;3(1):44–55. 10.1177/193229680900300106 19444330 PMC2681269

[8] Sorensen JT. A physiologic model of glucose metabolism in man and its use to design and access improved insulin therapies for diabetes. Massachussets Institute of Technology; 1985.

[9] Bergman RN, Ider YZ, Bowden CR, Cobelli C. Quantitative estimation of insulin sensitivity. American Journal of Physiology. 1979;236:667–677. 10.1152/ajpendo.1979.236.6.E667 443421

[10] Panunzi S, Palumbo P, De Gaetano A. A discrete single delay model for the intra-venous glucose tolerance test. Theoretical Biology and Medical Modelling. 2007;4(35). 10.1186/1742-4682-4-35 17850652 PMC2072949

[11] Panunzi S, De Gaetano A, Mingrone G. Advantages of the single delay model for the assessment of insulin sensitivity from the intravenous glucose tolerance test. Theoretical Biology and Medical Modelling. 2010;7(1):9 10.1186/1742-4682-7-9 20298586 PMC2858103

[12] De Fronzo RA, Tobin JD, Andres R. Glucose clamp technique: a method for quantifying insulin secretion and resistance. American Journal of Physiology. 1979;237(3). 10.1152/ajpendo.1979.237.3.E214 382871

[13] De Gaetano A, Arino O. Mathematical modelling of the intravenous glucose tolerance test. Journal of Mathematical Biology. 2000;40:136–168. 10.1007/s002850050007 10743599

[14] De Gaetano A, Panunzi S, Matone A, Samson A, Vrbikova J, Bendlova B, et al Routine OGTT: a robust model including incretin effect for precise identification of insulin sensitivity and secretion in a single individual. PLoS One. 2013;29(8(8)). 10.1371/journal.pone.0070875 PMC3756988 24009656

[15] Palumbo P, De Gaetano A. An islet population model of the endocrine pancreas. Journal of Mathematical Biology. 2010;61 10.1007/s00285-009-0297-0 19756607

[16] Grodsky GM. A Threshold Distribution Hypothesis for Packet Storage if Insulin and Its Mathematical Modeling. Journal of Clinical Investigation. 1972;51 10.1172/JCI107011 4559946 PMC292361

[17] De Gaetano A, Gaz C, Palumbo P, Panunzi S. A Unifying Organ Model of Pancreatic Insulin Secretion. PLoS ONE. 2015;10(11):1–34. 10.1371/journal.pone.0142344 PMC4640662 26555895

[18] Palumbo P, Panunzi S, De Gaetano A. Qualitative behavior of a family of of delay-differential equations models of the glucose-insulin regulatory system. Discrete and Continuous Dynamical Systems—B. 2007;7(2):399–424.

[19] Simon C, Brandenberger G, Follenius M. Ultradian Oscillations of Plasma Glucose, Insulin and C-Peptide in Man during Continuous Enteral Nutrition. Journal of Clinical Endocrinology and Metabolism. 1987;. 10.1210/jcem-64-4-669 3102544

[20] Sturis J, Pugh WL, Tang J, Ostrega DM, Polonski JS, Polonski KS. Alterations in pulsatile insulin secretion in the Zucker diabetic fatty rat. American Journal of Physiology. 1994;267:E250–E259. 10.1152/ajpendo.1994.267.2.E250 8074204

[21] Pørksen N, Juhl C, Hollingdal M, Pincus SM, Sturis J, Veldhuis JD, et al Concordant induction of rapid in vivo pulsatile insulin secretion by recurrent punctuated glucose infusions. American Journal of Physiology-Endocrinology and Metabolism. 2000;278. 10.1152/ajpendo.2000.278.1.E162 10644551

[22] Mari A, Tura A, Gastaldelli A, Ferrannini E. Assessing insulin secretion by modeling in multiple-meal tests: role of potentiation. Diabetes. 2002;51 10.2337/diabetes.51.2007.S221 11815483

[23] Toschi E, Camastra S, Sironi AM, Masoni A, Gastaldelli A, Mari A, et al Effect of acute hyperglycemia on insulin secretion in humans. Diabetes. 2002;51(Suppl.1):S130–S133. 10.2337/diabetes.51.2007.S130 11815471

[24] Gaz C, De Gaetano A, Manes C, Palumbo P, Borri A, Panunzi S. Effective Control of Glycemia using a Simple Discrete-delay Model. IFAC-PapersOnLine. 2017;50(1):13526–13531. 10.1016/j.ifacol.2017.08.2345.

[25] Martin EC, Yates JWT, Ogungbenro K, Aarons L. Choosing an optimal input for an intravenous glucose tolerance test to aid parameter identification. Journal of Pharmacy and Pharmacology. 2017;69(10):1275–1283. 10.1111/jphp.12759 28653461

[26] Hahn RG, Ljunggren S, Larsen F, Nystrom T. A simple intravenous glucose tolerance test for assessment of insulin sensitivity. Theoretical Biology and Medical Modeling. 2011;8:12 10.1186/1742-4682-8-12 PMC3113339 21535887

[27] Polyzogopoulou EV, Kalfarentzos F, Vagenakis AG, Alexandrides TK. Restoration of euglycemia and normal acute insulin response to glucose in obese subjects with type 2 diabetes following bariatric surgery. Diabetes. 2003;52(5):1098–1103. 10.2337/diabetes.52.5.1098 12716738

[28] Ward GM, Walters JM, Barton J, Alford FP, Boston RC. Physiologic modeling of the intravenous glucose tolerance test in type 2 diabetes: a new approach to the insulin compartment. Metabolism—Clinical and Experimental. 2001;50(5):512–519. 10.1053/meta.2001.21029 11319711

[29] Othman NA, Docherty PD, Krebs JD, Bell DA, Chase JG. The Need to Calculate Target Glucose Levels When Measuring Changes in Insulin Sensitivity During Interventions for Individuals With Type 2 Diabetes. Journal of Diabetes Science and Technology. 2018;12(3):665–672. 10.1177/1932296817750402 29295634 PMC6154237

[30] Pedersen MG, Corradin A, Toffolo GM, Cobelli C. A subcellular model of the glucose-stimulated pancreatic insulin secretion. Philosophical Transactions of the Royal Society A. 2008;366:3525–3543. 10.1098/rsta.2008.0120 18653438

[31] Breda E, Cavaghan M, Toffolo G, Polonsky K, Cobelli C. Oral glucose tolerance test minimal model indexes of beta-cell function and insulin sensitivity. Diabetes. 2001;50(1):150–8. 10.2337/diabetes.50.1.150 11147781

[32] Dalla Man C, Rizza RA, Cobelli C. Meal simulation model of the glucose insulin system. IEEE Transactions on Biomedical Engineering. 2007;54(10):1740–1749. 10.1109/TBME.2007.893506 17926672

[33] Dalla Man C, Micheletto F, Lv D, Kovatchev B, Cobelli C. The UVA/PADOVA Type I Diabetes Simulator: New Features. Journal of Diabetes Science and Technology. 2014;. 10.1177/1932296813514502 PMC4454102 24876534

[34] Bergman RN, Phillips LS, Cobelli C. Physiologic evaluation of factors controlling glucose tolerance in man: measurement of insulin sensitivity and beta-cell glucose sensitivity from the response to intravenous glucose. Journal of Clinical Investigation. 1981;68(6):1456–1467. 10.1172/JCI110398 7033284 PMC370948

[35] Mari A. Mathematical modeling in glucose metabolism and insulin secretion. Current Opinion in Clinical Nutrition and Metabolic Care. 2002;5(5):495–501. 10.1097/00075197-200209000-00007 12172472

[36] Mari A, Schmitz O, Gastaldelli A, Oestergaard T, Nyholm B, Ferrannini E. Meal and oral glucose tests for assessment of beta -cell function: modeling analysis in normal subjects. Americal Journal of Physiology Endocrinology and Metabolism. 2002;283(6):E1159–1166. 10.1152/ajpendo.00093.2002 12388151

[37] Toffolo G, Campioni M, Basu R, Rizza RA, Cobelli C. A minimal model of insulin secretion and kinetics to assess hepatic insulin extraction. American Journal of Physiology Endocrinology and Metabolism. 2006;290(1):E169–E176. 10.1152/ajpendo.00473.2004 16144811

[38] De Gaetano A, Hardy T, Beck B, Abu-Raddad E, Palumbo P, Bue-Valleskey J, et al Mathematical models of diabetes progression. American Journal of Physiology Endocrinology and Metabolism. 2008;295(6):E1462–79. 10.1152/ajpendo.90444.2008 18780774

[39] Toghaw P, Matone A, Lenbury Y, De Gaetano A. Bariatric surgery and T2DM improvement mechanisms: a mathematical model. Theoretical Biology and Medical Modelling. 2012;. 10.1186/1742-4682-9-16 22587410 PMC3586953

[40] Sturis J, Van Cauter E, Blackman JD, Polonski KS. Entrainment of Pulsatile Insulin Secretion by Oscillatory Glucose Infusion. Journal of Clinical Investigation. 1991;87:439–445. 10.1172/JCI115015 1991830 PMC295095

